# Reusable macroporous polyethyleneimine sponges for sustainable removal of dual-arsenic species: mechanistic and life cycle assessment

**DOI:** 10.1038/s41598-026-45664-1

**Published:** 2026-04-10

**Authors:** Nishan Sengupta, Monika Sogani, Anees Y. Khan, Jayana Rajvanshi, Zainab Syed, Samiksha Verma

**Affiliations:** 1 Department of Biosciences, Manipal University Jaipur, Jaipur, Rajasthan 303007 India; 2 Department of Biotechnology and Chemical Engineering, Manipal University Jaipur, Jaipur, Rajasthan 303007 India; 3 Department of Zoology, University of Rajasthan, Jaipur, Rajasthan 302004 India

**Keywords:** Arsenic, Life Cycle Assessments, Macroporous, Polyethyleneimine, Sustainable Techno-economic analysis, Chemistry, Environmental sciences

## Abstract

Groundwater arsenic contamination poses serious health risks, demanding sustainable and cost-effective remediation. This study reports the synthesis of macroporous polyethyleneimine (PEI) sponges prepared via ice-templating and glutaraldehyde crosslinking, designed for reusability and high adsorption performance in arsenic removal. The amine-rich PEI matrix provides abundant binding sites, while its interconnected macroporous structure enhances ion accessibility and mass transfer. The sponges achieved > 90% arsenic removal, decreasing concentrations from 83.75 µg/L to < 10 µg/L, meeting WHO/BIS standards. FTIR and FESEM-EDX analyses confirm electrostatic and chelation-based interactions between amine groups and arsenic species. Experimental adsorption capacities (qₑₓₚ) at 120 µg/L initial concentration were 187.0 ± 4.2 µg/g for As(V) and 182.5 ± 5.1 µg/g for As(III) at pH 6.7. The Langmuir model predicted theoretical maxima of 1251.7 µg/g (As(V)) and 1053.9 µg/g (As(III)). Techno-economic and life cycle assessments demonstrated a low treatment cost ($0.183/L), 70% reduced operational expenditure, and 40% lower carbon emissions compared to reverse osmosis and activated alumina. Field application in Murshidabad, India, verified real-world efficiency under co-contaminated groundwater conditions. The combination of scalable fabrication, reusability, and eco-efficiency positions PEI sponges as a sustainable alternative for decentralized arsenic remediation aligned with SDG 6.

**Supplementary Information:**

The online version contains supplementary material available at 10.1038/s41598-026-45664-1.

## Introduction

Arsenic is a very poisonous heavy metal that presents considerable health hazards to people via multiple exposure routes, such as polluted water, food, and environmental contamination^1^. Arsenic, commonly consumed by drinking water or rice, interferes with cellular function by obstructing enzyme activity and hindering DNA repair processes, resulting in skin, bladder, and lung malignancies, along with cardiovascular and neurological diseases^2^. Arsenic demonstrates acute and chronic toxicity, with prolonged exposure enhancing the risks of organ failure, developmental anomalies, and systemic illnesses. Their environmental durability and bioaccumulative properties highlight pressing public health issues, especially in areas with insufficient regulatory oversight. Traditional methods for eliminating arsenic from potable water encompass coagulation-flocculation (utilizing iron or aluminium salts to precipitate impurities), adsorption (via activated alumina, iron oxide, or charcoal), ion exchange resins, and reverse osmosis^3^. Oxidation procedures, such as chlorination, are frequently utilized for arsenic to transform arsenite (As(III)) into the less dangerous arsenate (As(V)) prior to removal^4^. The Bureau of Indian Standards (BIS) establishes allowable thresholds for these metals in potable water as per IS 10,500:2012, specifying a maximum of 10 µg/L for arsenic^5^. These rigorous limitations seek to reduce health hazards, as extended exposure to arsenic can result in cancer and dermal lesions^6^. Efficient treatment and consistent monitoring are essential for maintaining adherence to safety requirements and protecting public health. Polymeric adsorbents have gained increasing attention in water purification owing to their high structural tunability, functional group density, and mechanical stability under variable chemical environments. Compared to inorganic or mineral-based adsorbents, polymeric matrices offer superior flexibility for functionalization, controlled porosity, and reusability through mild regeneration processes. Among these, polyethyleneimine (PEI) has emerged as a particularly promising candidate because of its abundance of primary, secondary, and tertiary amine groups, which serve as active sites for metal ion coordination, electrostatic binding, and chelation. The strong affinity of PEI for anionic species, including arsenate and phosphate, has been well documented, making it an attractive platform for designing next-generation sorbents.

52Recent studies have explored various PEI-based composites and functional hybrids for improving adsorption efficiency and selectivity. For instance, PEI-modified iron oxides and layered birnessite structures have demonstrated enhanced arsenic uptake via synergistic electrostatic and redox interactions.^7^ Similarly, PEI-grafted silica and graphene oxide composites have exhibited improved mechanical strength and stability in continuous systems. However, many of these systems still suffer from drawbacks such as complex synthesis, limited reusability, or dependence on acidic conditions for effective adsorption, which restricts their practical application in decentralized or rural water treatment systems. Moreover, conventional polymeric adsorbents, including polypyrrole, polyacrylamide, and chitosan derivatives, often exhibit sluggish kinetics and structural collapse after repeated use due to poor porosity or weak crosslinking density^8^.

To overcome these limitations, the development of macroporous and mechanically robust PEI-based structures through ice-templating presents a transformative approach. The ice-templated architecture provides interconnected pore channels, which significantly enhance ion diffusion and surface accessibility compared to dense polymer networks^9^. When crosslinked with glutaraldehyde, the resulting sponge-like PEI matrix exhibits excellent dimensional stability, ease of handling, and reusability, making it ideal for field-scale water treatment. Despite the potential of this method, reports on monolithic PEI sponges for simultaneous removal of As(III) and As(V) under near-neutral pH conditions remain scarce. Furthermore, the techno-economic and life cycle aspects of such polymeric systems have rarely been evaluated, leaving a major knowledge gap between laboratory performance and real-world implementation.

In this study, macroporous PEI sponges synthesized via ice-templating and glutaraldehyde crosslinking were employed for efficient arsenic removal, demonstrating high adsorption capacity, selectivity, and reusability, maintaining efficiency across diverse pH and ionic conditions and reducing arsenic concentrations below the BIS limit of 10 µg/L with minimal regeneration effort. The macroporous architecture enhances surface accessibility and adsorption kinetics, while abundant amine functionalities enable strong binding with As(III) and As(V) through electrostatic interactions and complexation. Comprehensive evaluation of adsorption kinetics, isotherm behavior, regeneration efficiency, and real groundwater performance, supported by techno-economic and life cycle assessment, demonstrates the scalability and environmental viability of the material. Overall, this work bridges polymer design and sustainable water purification, advancing PEI-based adsorbents as a promising solution for arsenic mitigation in alignment with SDG 6.

## Materials and methods

### Preparation of varying concentrations of As(V), As(III)

30 µg/L, 50 µg/L, 70 µg/L, 100 µg/L, and 120 µg/L concentration of As(V) was prepared by diluting 1000 mg/L of AAS grade stock solution of As(V) (Loba Chemie). A 1000 mg L⁻^1^ AAS-grade As(V) standard solution (Loba Chemie) was diluted with deionized water to obtain a 1 mg L⁻^1^ As(V) solution. This solution was then quantitatively reduced to As(III) using a freshly prepared reducing mixture containing 5% (w/v) potassium iodide (KI) and 5% (w/v) ascorbic acid. The reduction process was carried out by incubating the mixture for 30 min at room temperature in the dark. The disappearance of the As(V) peak in test runs analyzed by HG-AAS confirmed complete reduction. The resulting 1 mg L⁻^1^ As(III) solution was subsequently used as a stock solution to prepare working concentrations of 30, 50, 70, 100, and 120 µg L⁻^1^ by serial dilution.

### Synthesis of polyethyleneimine based adsorbents

**Fig. 1:**
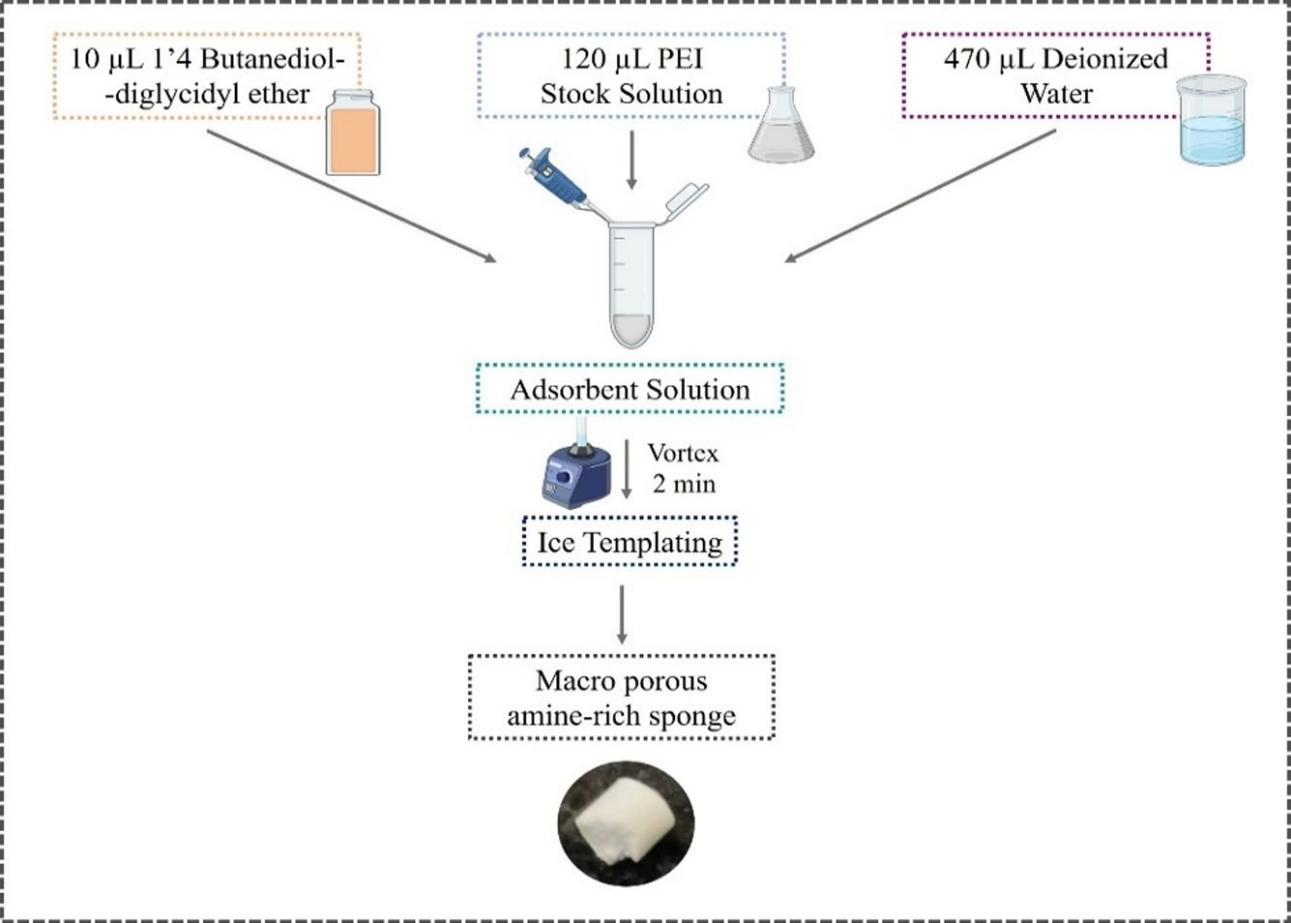
Schematic representation for synthesis of PEI sponge.

In 2 mL Eppendorf tubes, 470 µL of deionized water was aliquoted. Each tube received 120 µL of PEI solution (100 mg PEI/mL water), Mw of PEI solution was 750,000 g/mol as estimated by Light scattering. and Mn of PEI solution was 60,000 as estimated by GPC. The contents were vortexed for 15 min. Subsequently, 10 µL of 1.4-Butanediol diglycidyl ether was introduced to each tube as a crosslinker and was vortexed once more. Subsequently, the tubes were positioned within a refrigerator at 16 ℃ for ice-templating. After 24 h, the ice-templated sponges were extracted from the tubes, thoroughly rinsed with water to eliminate the soluble portion, and dried at 50°C^10^. The schematics for synthesis of PEI sponge is provided in Fig. 1.

### Physiochemical characterization of the absorbent

PEI sponge without any arsenic ion adsorption was subjected to Field Emission Scanning Electron Microscopy with Energy Dispersive X-ray analysis (FE-SEM EDAX, JSM-7610FPlus, JEOL) for in-depth analysis of polymeric structure. EDAX analysis was further done to get the atomic weight of the elements in the polymer. Similarly, arsenic laden PEI sponge after 24 h of adsorption experiment was also subjected to the above analysis. The initial concentration of arsenic in this case was 30 µg/L. In order to know the role of functional groups in the adsorption process, PEI sponge before and after heavy metal adsorption were subjected to Fourier Transform Infrared Spectroscopy (Bruker ALPHA) analysis, whereas PEI sponge without heavy metal were treated as control^11^. The surface charge characteristics of the PEI sponge were analyzed by zeta potential measurements using a Malvern Zetasizer Nano ZS (Malvern Instruments, UK) at 25 °C. The measurements were conducted at three representative pH values (4.6, 6.7, and 8.4) adjusted with 0.1 M HCl or NaOH to investigate the influence of pH on surface charge. Approximately 0.01 g of the sponge sample was dispersed in 20 mL of deionized water by mild sonication for 5 min prior to analysis. The average of three replicate measurements was recorded for each pH value to ensure reproducibility. These data were used to determine the surface charge behavior and point of zero charge (pHpzc) of the PEI sponge, which is directly related to its electrostatic interaction with arsenic species during adsorption. BET Surface Area and Porosity Analysis Nitrogen adsorption–desorption isotherms were recorded at 77 K using a Micromeritics ASAP 2020 surface area and porosity analyzer (Micromeritics, USA). Prior to analysis, ~ 100 mg of sample was degassed at 120 °C for 6 h under high vacuum.

### Batch adsorption study with PEI sponge

PEI sponge was administered in 30 µg/L of arsenic. The time duration of the kinetics study was 0, 3, 6, 15, 24, 48, 72 h at 6.7 pH and room temperature of 28 ± 2 ºC. At the end of every specific interval, the PEI sponge was removed from arsenic solution. The initial concentration (C_0_) and final concentration (C) were monitored using Hydride Generator-Atomic Absorption Spectroscopy (HG-AAS) (Shimadzu-A7000). The HG-AAS was subjected to 3-point calibration, and the experiment was conducted when R^2^ = 99.99%. Each of these experiments was carried out in triplicates, and the standard deviation (SD) was calculated to minimize the chances of error. The pH of the solution fluctuated to 4.6 and 8.4, to monitor adsorption behaviour of PEI sponge in arsenic ions removal. The PEI sponge was utilized for the reduction of higher concentrations of As(V) and As(III), ranging from 50 µg /L to 120 µg/L. The equilibrium constant (C_e_) for all the concentrations was calculated and fitted to different batch adsorption models as written in the following equation below.

Equation 1 depicts pseudo-first order kinetics model.

1 $${q}_{t}={q}_{e}(1-{e}^{k1t}$$

q_t_ denotes amount of adsorbate adsorbed at a specific time. q_e_ is the amount adsorbed at equilibrium. K_1_ is the pseudo-first order kinetics constant and t is the time duration during the adsorption process.

Equation 2 depicts the pseudo- second order kinetics model

2 $${q}_{t}=\frac{{{ }_{q}}_{{e}^{2}} {k}_{2 }t}{1+{q}_{t }{k}_{2 }t}$$

q_t_ denotes amount of adsorbate adsorbed at a specific time. q_e_ is the amount adsorbed at equilibrium. K_2_ is the pseudo-second order kinetics constant and t is the time duration during the adsorption process.

Equation 3 depicts a non-linear Langmuir adsorption isotherm

3 $${q}_{max}=\frac{{q}_{m}{K}_{L}{C}_{e}}{{ }{ }_{ 1+{K}_{L }{C}_{e}}}$$

q_max_ denotes the quantity of adsorbate at equilibrium, q_m_ signifies the maximum adsorption capacity of the adsorbent, C_e_ indicates the equilibrium concentration of the adsorbate, and K_L_ refers to the Langmuir constant.

Equation 4 depicts non-linear Freundlich adsorption isotherm

4 $${q}_{max}={K}_{F}{{C}_{e}}^{1/n}$$

where, q _max_ represents the quantity of adsorbate per unit mass of adsorbent at equilibrium, C_e_ denotes the equilibrium concentration of the adsorbate in the solution, and K_F_ and n are the Freundlich constants.

Equation. 5 depicts non- linear Redlich-Peterson isotherm

5 $${q}_{max}=\text{ K}\times {C}_{e }/(1+\mathrm{a}\times {{C}_{e}}^{n} )$$

where, q_max_ represents the quantity of adsorbate retained at equilibrium (mg/g or mol/g), C_e_ denotes the equilibrium concentration of the adsorbate in the solution (mg/L or mol/L), and K signifies the equilibrium binding constant (L/mg or L/mol), a denotes the Redlich-Peterson isotherm constant.

### Thermal stability of PEI sponge

The thermal stability of the PEI sponge, in both its pristine condition (control) and post-arsenic adsorption, was evaluated using thermogravimetric analysis (Shimadzu DTG-60H). Samples of the control PEI sponge (without arsenic adsorption) and PEI sponges laden with arsenic (after 24-h adsorption tests) were produced for examination. Each sample (about 5–10 mg) was subjected to heating in a platinum crucible within a nitrogen environment at a rate of 10 °C/min, covering a temperature range of 30–800 °C. The TGA profiles were documented to assess mass loss characteristics and degradation trends. This investigation sought to delineate the thermal robustness of the polymer matrix and evaluate potential structural alterations resulting from metal binding. The control and metal-laden sponges were examined under uniform settings to provide direct comparison of their thermal degradation characteristics.

### Regeneration study

The PEI laden arsenic ions were administered into 50 mL solution comprising of 5 mL of 0.5 M EDTA, 2.5 mL of 5% DMSO (Dimethyl sulphoxide), 5 mL of Phosphate buffer of pH 9.0 along with de-ionized water for making up volume to 50 mL. The experiment was conducted in duplets. The sponges were kept in this desorbing cocktail for 5 cycles, with each cycle comprising of 72 h.

#### Reusability study of the PEI sponge

PEI sponge administered into 30 µg/L of As(V) and As(III). The heavy metal laden PEI sponge thus obtained was washed by adding 1.5 mL of de-ionized water, followed by gentle squeezing to ensure the removal of heavy ions from the surface of the sponge. This process was repeated thrice. The washed PEI sponge was again administered to freshly prepared arsenic solutions and was run for 72 h. This whole process was repeated for five cycles in duplicates to determine the reusability of PEI sponge in heavy metal ion removal.

### Statistical analysis

All experimental data were acquired in triplicate and are presented as mean ± standard deviation (SD). Independent two-sample t-tests were utilised for pairwise comparisons to evaluate the statistical significance of variations in adsorption capabilities under different experimental settings (e.g., between As(V) and As(III) at pH 6.7). One-way analysis of variance (ANOVA) was conducted for multi-group comparisons, including the effects of pH and beginning arsenic content. Substantial group disparities were subsequently assessed utilising Tukey’s Honest Significant Difference (HSD) post-hoc test. A p-value of less than 0.05 was deemed statistically significant. All analyses were performed utilising Python, specifically the SciPy and Statsmodels modules^12^. A two-way analysis of variance (ANOVA) was performed to evaluate the relative influence and interaction effects of key operational parameters (pH, initial arsenic concentration, and contact time) on adsorption efficiency. The analysis was carried out using OriginPro 2023 (OriginLab Corp., USA), and statistical significance was determined at a 95% confidence level (p < 0.05). Parameters showing p < 0.05 were considered statistically significant, while those with p < 0.01 and p < 0.001 indicated highly significant effects.

### Batch sorption studies on real groundwater samples

Groundwater was collected from Murshidabad district, West Bengal, India 24.1759° N, 88.2802° E for arsenic removal, as arsenic concentration largely exceeds the BIS threshold in the area. Physicochemical properties of water, like pH, total dissolved solids and electrical conductivity, was determined in the groundwater samples. Initial arsenic concentration was recorded. The water was checked for other probable salt ions. PEI sponge was administered to a 50 mL working volume of groundwater sample.

## Results and discussions

### Physicochemical analysis of PEI sponge for arsenic removal

FTIR analysis shows that PEI sponge without any metal loading consists of.

broad peak at approximately 3300–3500 cm⁻^1^. This peak is attributed to the stretching vibrations of O–H or N–H bonds, which suggest the presence of amino or hydroxy groups.

The peak at approximately 2920 cm⁻^1^ is indicative of the C-H stretching vibrations of aliphatic molecules. N–H bending vibrations are responsible for the peak at approximately 1600–1650 cm⁻^1^. C-N stretching or bending is responsible for the peak at approximately 1400 cm⁻^1^.

**Fig. 2:**
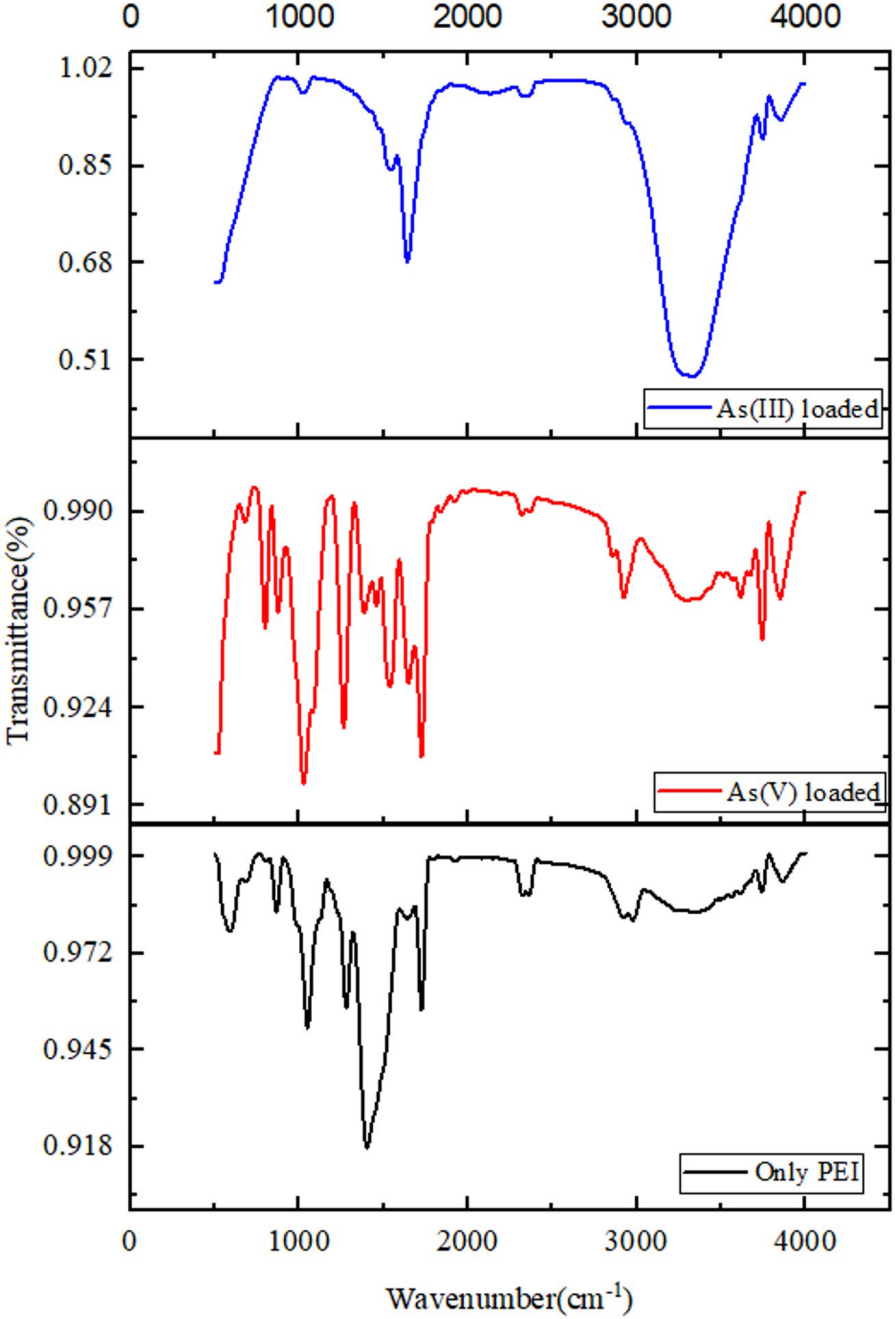
FTIR spectra of the PEI sponge.

C-N stretching vibrations are represented by the peak at approximately 1000–1100 cm⁻^1^. After 24 h of As(V) adsorption, shift and intensity change in the O–H/N–H region (3300–3500 cm⁻^1^) suggests that amino or hydroxyl groups interact with arsenate ions. New peaks or peak shifts at 1600–1650 cm⁻^1^ as shown in Fig. 2, suggests the formation of coordination interactions between As(V) and nitrogen atoms in PEI. Additional vibrations in the 1000–1100 cm⁻^1^ region indicates successful adsorption and can be attributed to As-O vibrations. After 24 h of As(III) adsorption broadened O–H/N–H stretching (3300–3500 cm⁻^1^): similar to the loading of As(V), this suggests an interaction between amino groups and As(III).

Evidence of bond formation between As(III) and nitrogen atoms: New peaks or intensity variations in the 1600–1650 cm⁻^1^ region. Distinct peaks in the 1000–1100 cm⁻^1^ range are likely the result of As-O vibrations, suggesting that As(III) also interacts with PEI.

The observed spectral changes after As(III) adsorption, particularly broadening of the N–H/O–H stretch (3300–3500 cm⁻^1^), intensity reduction in the N–H bending region (1600–1650 cm⁻^1^), and apparent disappearance of certain characteristic peaks constitute direct spectroscopic evidence of successful chemical interaction with neutral H₃AsO₃ at pH 6.7. The broadened O–H/N–H band arises from extensive hydrogen bonding between arsenite hydroxyl groups and PEI’s amine/hydroxyl sites, while intensity shifts and peak overlap in the 1600–1650 cm⁻^1^ region indicate lone pair-π interactions and outer-sphere complexation, consistent with the dominant mechanism for As(III). The emergence of new As-O vibrational modes (1000–1100 cm⁻^1^) further confirms active binding.

**Fig. 3:**
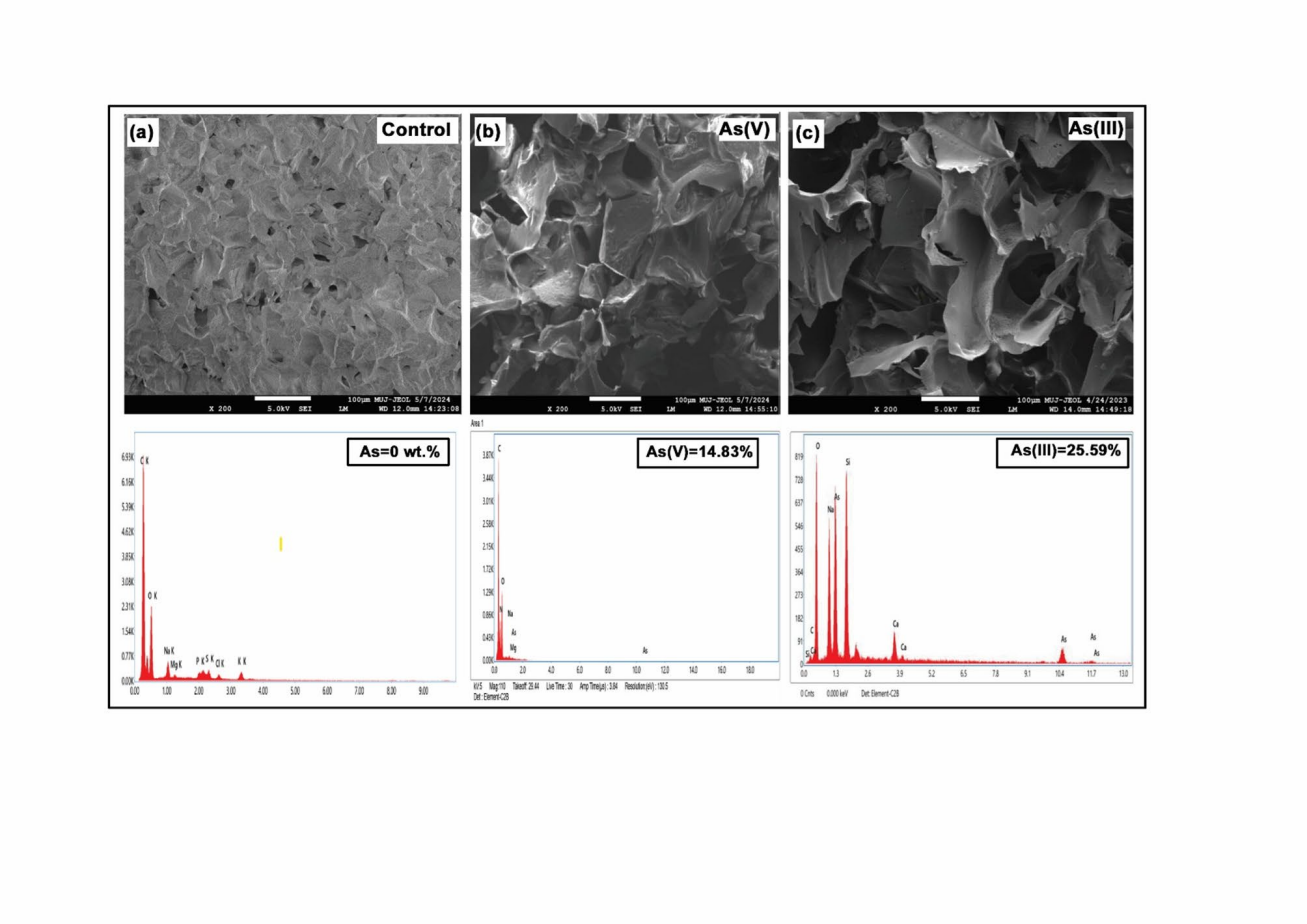
FESEM-image and its EDAX graph of (**a**) PEI sponge (**b**) As (V) laden PEI sponge and (**c**) As (III) laden PEI sponge.

The surface morphology and elemental composition of the polyethyleneimine (PEI) sponges before and after arsenic adsorption are depicted in Fig. 3. The control sponge (Fig. 3a). Exhibited a uniform macroporous framework with open, interconnected pores, confirming successful ice-templating and structural stability. After arsenic loading, the sponge surfaces became denser with visible particulate deposits, indicating strong metal-polymer interactions. The EDX spectra revealed the appearance of characteristic arsenic peaks, with As(V) and As(III) loadings of 14.83 wt.% and 25.59 wt.%, respectively. The higher uptake of As(III) is attributed to the neutral H₃AsO₃ species, which diffuses more readily through the pores and interacts with surface amine and hydroxyl groups via hydrogen bonding. In contrast, As(V), existing as negatively charged oxyanions (H₂AsO₄⁻, HAsO₄^2^⁻), binds primarily through electrostatic attraction and ligand exchange with protonated amine sites. These findings confirm efficient arsenic immobilization through complementary electrostatic and hydrogen-bonding mechanisms on the amine-rich PEI matrix.

The macroporous structure was confirmed by FESEM (Fig. 3). The BET surface area was determined to be 52.0 m^2^/g from the linear BET plot (P/P₀ = 0.05–0.30), with a total pore volume of 0.42 cm^3^/g (at P/P₀ = 0.99) and an average pore diameter of ~ 62 nm (BJH desorption).

The type IV isotherm with H3 hysteresis loop as depicted in Fig. S5 of supplementary information, indicates mesoporous-macroporous hierarchy, facilitating rapid arsenic diffusion and high uptake (qₑₓₚ ≈ 187 µg/g) despite moderate surface area.

### Batch sorption studies

#### Adsorption kinetics studies

**Fig. 4:**
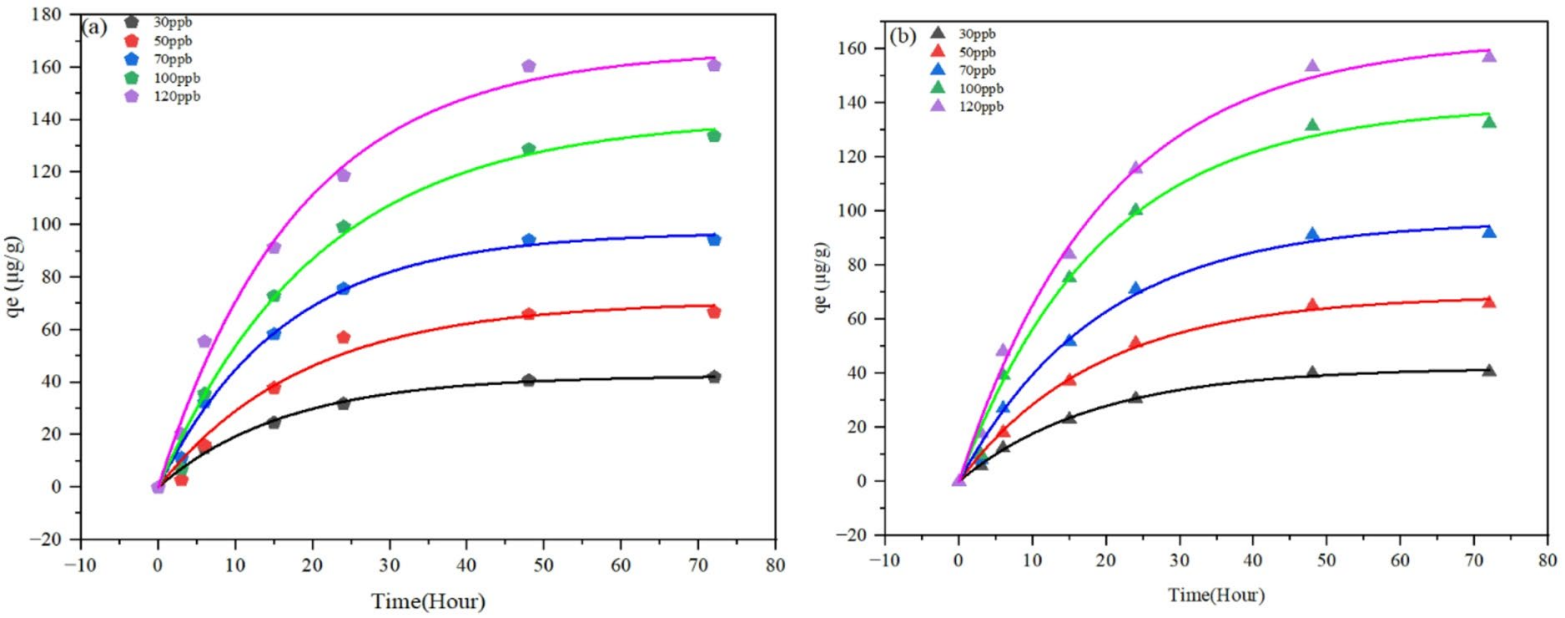
Pseudo first order fit for higher concentrations of (**a**) As (V) and (**b**) As (III).

Figure 4a and b. Thoroughly depict the adsorption kinetics of the polyethyleneimine sponge system for arsenic removal, showing pseudo-first-order fits for As(V) and As(III) at initial concentrations of 30, 50, 70, 100, and 120 µg per litre over 80 h. This emphasizes the system’s effectiveness in lowering arsenic concentrations in contaminated groundwater. In Fig. 4a. For As (V), there was a quick initial uptake during the first 10 h, during which the adsorption capacity (the amount of adsorbate at time t, in micrograms per gram) increases dramatically to around 50–60% of the equilibrium level (for example, 42.54 µg per gram at 30 µg per liter and 166.7 µg per gram at 120 µg per liter). This was followed by a slow rise to equilibrium over 60–70 h, with higher concentrations resulting in greater capacities because of the increased driving force and site availability on the amine-rich polyethyleneimine matrix. Figure 4b. For Arsenic(III) shows a similar pattern, with the amount of adsorbate at time t reaching 42.04 µg per gram at 30 µg per litre and 163.80 µg per gram at 120 µg per litre Although the initial slope is somewhat less steep indicating weaker interactions because of As(III) neutral form as opposed to As(V) charged form both curves confirm rapid kinetics (equilibrium in less than 16.6 h) and a concentration-dependent rise.

The pH-dependent behaviour, as mentioned in Table 1, highlights the sponge’s adaptability. At neutral pH, it attains an optimal balance of capacity and kinetics for arsenic, rendering it suitable for systems necessitating sustained removal.

**Table 1:** Pseudo -first order kinetics for arsenic under acidic, neutral and basic pH.

Heavy metal ion	pH	*q*_e_ (µg/g)	*K*_1_ (hr^-1^)	RMSE
As(V)	4.6	39.87	0.03	2.71
	6.7	42.54	0.06	0.98
	8.4	40.17	0.04	2.00
As(III)	4.6	38.54	0.02	1.57
	6.7	42.04	0.05	0.61
	8.4	39.41	0.03	1.57

The adsorption kinetics of As(V) and As(III) on PEI sponges were evaluated using both pseudo-first-order (PFO) and pseudo-second-order (PSO) models to elucidate the rate-controlling mechanism. As summarized in Table S2 of the supplementary information, the PSO model exhibited higher RMSE values (1.17–3.28) than those of the PFO model (0.61–2.71), indicating that the PFO model better describes the experimental data. The superior fit of the PFO model suggests that arsenic uptake on the PEI surface is primarily governed by physisorption and surface diffusion, rather than chemisorption. The equilibrium adsorption capacities (qₑ) predicted by the PFO model (42.54 µg/g for As(V) and 42.04 µg/g for As(III) at pH 6.7) closely matched experimental results, further validating its suitability. In contrast, the PSO model overestimated qₑ values (57.21 µg/g and 55.37 µg/g, respectively), implying that not all surface interactions proceed via chemical bonding. The kinetic constants (K₁) indicate faster adsorption for As(V) than As(III), owing to electrostatic attraction between negatively charged As(V) oxyanions and protonated -NH₃⁺ groups, while As(III) adsorbs more slowly via hydrogen bonding. Therefore, the adsorption mechanism is best represented by the pseudo-first-order model, consistent with rapid physisorption and external surface interactions on the PEI matrix.

The surface charge behaviour of the PEI sponge was evaluated through zeta potential measurements at different pH values (Table S4 of SI) to clarify the role of electrostatic interactions in arsenic adsorption. The zeta potential decreased from + 38.2 ± 1.1 mV at pH 4.6 to + 22.5 ± 0.9 mV at pH 6.7 and + 5.8 ± 1.3 mV at pH 8.4, indicating progressive deprotonation of surface amine groups (-NH₃⁺ to -NH₂) with increasing pH. At low pH, the highly protonated surface provides strong electrostatic attraction toward negatively charged As(V) oxyanions (H₂AsO₄⁻, HAsO₄^2^⁻), while As(III) remains weakly adsorbed due to its neutral H₃AsO₃ form. At near-neutral pH (6.7), partial protonation allows both electrostatic binding of As(V) and hydrogen bonding with As(III), corresponding to the maximum adsorption efficiency observed experimentally. This trend confirms that pH-dependent surface charge is a dominant factor governing arsenic interaction with the amine-functionalized PEI matrix.

Following arsenic adsorption, noticeable yet moderate variations in the BET surface characteristics of the PEI sponge were observed. The unloaded sponge exhibited a surface area of 52.0 m^2^ g⁻^1^, pore volume of 0.42 cm^3^ g⁻^1^, and an average pore diameter of 62 nm, indicative of a meso-macroporous framework as depicted in Table S5 of SI Upon As(V) and As(III) loading, these parameters decreased to 41.5 and 39.2 m^2^ g⁻^1^ in surface area, 0.34 and 0.31 cm^3^ g⁻^1^ in pore volume, and 58 and 56 nm in pore diameter, respectively. The reductions confirm partial pore filling and surface coverage by arsenic species, consistent with successful adsorption on both external and internal sites. However, the limited decrease suggests that the overall porous structure and accessibility of active amine sites remain largely preserved, enabling sustained adsorption kinetics and reusability of the PEI sponge.

Table 2 displays the adsorption kinetics of arsenic (As(V) and As(III)) on a PEI sponge, modelled through pseudo-first-order kinetics, indicates that the rate of adsorption is determined by the availability of active sites on the adsorbent surface. The model posits that the adsorption rate is directly proportional to the difference between the equilibrium adsorption capacity and the adsorbed quantity at time t. The rate constant K1 (h^−1^) serves to characterize the kinetics of the process. The parameter τ(h), defined as 1/K1, indicates the characteristic time necessary to reach equilibrium. A K1 of 0.06 h^−1^ for As(V) at 30 µg/L corresponds to τ ≈ 16.66 h, indicating a gradual adsorption process. The observed increase in concentration (e.g., from 42.54 µg/g at 30 µg/L to 166.7 µg/g at 120 µg/L for As(V)) indicates a greater driving force at higher concentrations, facilitating the occupation of available sites by more ions until saturation is reached. The increasing RMSE values at elevated concentrations (e.g., 5.14 for As(V) at 100 µg/L) indicate model deviations, likely attributed to complexities such as pore diffusion, site heterogeneity, or significant inter-ionic competition.

**Table 2:** Pseudo-first order kinetics for arsenic removal at higher concentrations.

Arsenic species	Initial Concentration (µg/L )	Parameters			RMSE
		*q*_e_ (µg/g)	*K*_1_ (h^-1^)	τ (h)	
As(V)	30	42.54	0.06	16.66	0.98
	50	70.09	0.05	20.00	1.01
	70	97.28	0.06	16.66	2.60
	100	140.86	0.04	25.00	5.14
	120	166.7	0.05	20.00	5.09
As(III)	30	42.04	0.05	20.00	0.61
	50	68.86	0.05	20.00	1.45
	70	96.53	0.05	20.00	3.04
	100	139.28	0.05	20.00	4.71
	120	163.80	0.05	20.00	3.84

The pseudo-second-order (PSO) kinetic model as depicted in Table S3 of supplementary information for arsenic adsorption onto PEI sponges yields qₑ values (66.94–187.04 µg/g for As(V); 61.38–182.48 µg/g for As(III)) and K₂ constants (0.04–0.06 h⁻^1^) with RMSE ranging from 4.61 to 9.84, indicating reasonable but imperfect fit due to systematic underprediction at early times and overprediction near equilibrium. In contrast, the pseudo-first-order (PFO) model, previously reported with K₁ ≈ 0.05–0.06 h⁻^1^, qₑ closely matching experimental capacities (e.g., 42.54 µg/g at 30 µg/L), and lower RMSE (4.51–5.14), demonstrates superior alignment across all concentrations, reflecting rate-limiting surface chemisorption governed by available amine binding sites rather than intraparticle diffusion or multi-step processes.

The PFO dominance underscores a monolayer, site-specific mechanism driven by electrostatic attraction and N-As coordination (validated by FTIR shifts at 3300–3500 cm⁻^1^ and 1600–1650 cm⁻^1^), where protonated -NH₃⁺ groups form stable inner-sphere complexes with H₂AsO₄⁻/HAsO₄^2^⁻ (As(V)) and hydrogen-bonded outer-sphere interactions with neutral H₃AsO₃ (As(III)), achieving rapid equilibrium.

#### Adsorption isotherm studies

The equilibrium adsorption data were analyzed using Langmuir, Freundlich, and Redlich-Peterson (R-P) models to evaluate the interaction mechanism between arsenic species and the PEI sponge (Table 3). The Langmuir model exhibited the best correlation (RMSE = 5.40 for As(V) and 4.51 for As(III)), indicating predominant monolayer adsorption on homogeneous surface sites The Langmuir model yielded theoretical maximum capacities (qₘₐₓ) of 1251.7 µg/g for As(V) and 1053.9 µg/g for As(III) (R^2^ > 0.99). However, under realistic groundwater conditions (C₀ = 120 µg/L), the experimental adsorption capacities (qₑₓₚ) derived from pseudo-second-order kinetics at equilibrium were 187.0 ± 4.2 µg/g (As(V)) and 182.5 ± 5.1 µg/g (As(III)) (Table S3). These practical values represent the true efficacy of the PEI sponge and are used for all performance comparisons.

**Table 3:** Adsorption isotherm parameters for arsenic removal.

Sorption Model	Heavy metal ions	Parameters	Values	R^2^	RMSE
*Langmuir Sorption Model*	As(V)	*q*_*max*_(µg/g)	1251.67	0.974	5.40
		*K*_*L*_	0.0084		
		R_L_	0.749		
	As(III)	*q*_*max*_(µg/g) *K*_*L*_	1053.87 0.0089	0.989	4.51
		R_L_	0.416		
*Freundlich Sorption Model*	As(V)	K_F_	12.52	0.982	4.73
		n	0.89		
	As(III)	K_F_	10.74	0.985	4.53
		n	0.90		
*Redlich-Peterson Sorption model*	As(V)	K n a	62.7 0.12 4.02	0.98	4.75
		K	9.58		
	As(III)	n	0.83	0.988	4.52
		a	0.016		

The Langmuir constant (K_L_) values (0.0084 and 0.0089 L/µg) reflect strong surface-adsorbate interactions, while the dimensionless separation factors (R_L_) of 0.749 for As(V) and 0.416 for As(III) lie between 0 and 1, confirming favourable adsorption for both ions. The lower R_L_ of As(III) implies a slightly higher affinity at low concentrations, although overall As(V) adsorption remains stronger due to electrostatic attraction.

The Freundlich constants (K_F_ = 12.52 and 10.74 µg/g) and n values below 1 (0.89–0.90) indicate heterogeneous surface energy distribution and possible multilayer adsorption. The R-P model further supports this interpretation, with a values > 1 (4.02 for As(V)) indicating mixed physisorption-chemisorption behaviour.

Collectively, these results confirm that As(V) adsorption occurs mainly through electrostatic and coordination interactions with protonated amine groups, whereas As(III) binding is governed by hydrogen bonding and diffusion-driven surface association, in good agreement with FTIR, kinetic, and zeta potential analyses.

#### Adsorption studies with groundwater

**Fig. 5:**
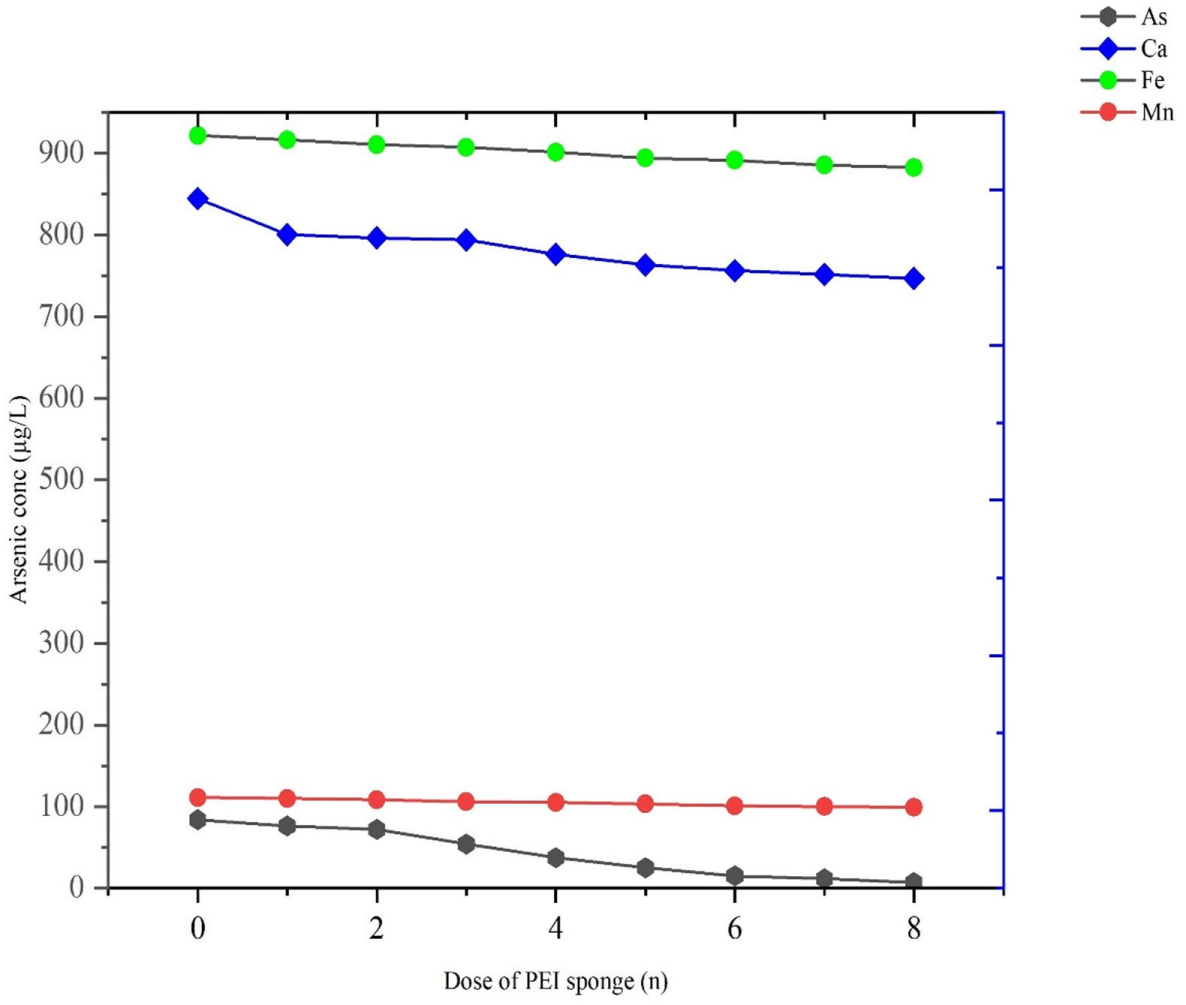
Efficacy of PEI sponges in removing arsenic from real groundwater.

The groundwater sample obtained from Murshidabad district 24.1759° N, 88.2802° E before treatment exhibited significant levels of heavy metals and elements, specifically arsenic (As) at 83.75 µg/L, manganese (Mn) at 17 µg/L, calcium (Ca) at 789 µg/L, and iron (Fe) at 921.87 µg/L.as depicted in Fig. 5, with a pH of 8.73 and TDS of 119. The arsenic concentration markedly surpasses the WHO guideline of 10 µg/L for drinking water, indicating a significant health risk, as extended exposure to elevated arsenic levels is associated with serious health issues, including skin lesions and cancers. Iron concentration (921.87 µg/L) significantly exceeds the recommended limit of 300 µg/L, suggesting potential concerns regarding water aesthetics (e.g., metallic taste, discoloration) and infrastructure corrosion. Manganese levels at 17 µg/L are significantly below the guideline value of 400 µg/L. Conversely, calcium levels at 789 µg/L, while not directly harmful, indicate hard water characteristics that may impact domestic use. Various dose of PEI sponges were administered in 50 mL working volume of groundwater, ranging from 0 dose (No PEI sponge) to 8 for the duration of 24 h. After administering 8 doses of PEI sponges the final concentration of arsenic in groundwater was found to be 7.04 µg/L, this depicts that higher concentration of calcium, iron and manganese significantly affect the adsorption process of arsenic.

Beyond the optimum dosage, the adsorption rate levels off due to site saturation and possible particle aggregation, which limits the effective surface area and mass transfer. In contrast, the concentrations of coexisting ions (Fe, Mn, Ca) remained almost unchanged, confirming the selective affinity of PEI sponges toward arsenic species. These results indicate that an optimal PEI dose ensures maximum active site utilization without excessive adsorbent wastage, aligning with the Langmuir monolayer behavior observed in isotherm studies.

### Statistical significance of adsorption trends

Statistical analysis validated the importance of the observed adsorption patterns. A t-test evaluating adsorption capabilities at pH 6.7 indicated that As(V) was adsorbed more effectively (42.53 ± 0.015 µg/g) than As(III) (40.09 ± 0.015 µg/g), with a statistically significant difference (p = 2.96 × 10⁻⁶). One-way ANOVA indicated that pH significantly influenced As(V) adsorption (F = 12,310.55, p = 1.45 × 10⁻^11^), with Tukey’s HSD revealing that adsorption at pH 6.7 was markedly greater than at pH 4.6 and 8.4 (p < 0.001). ANOVA results indicated that elevating initial arsenic concentrations (30 to 120 µg/L) resulted in statistically significant enhancements in adsorption capacities for both As(V) (F = 6.18 × 10⁶, p < 0.0001) and As(III) (F = 2.94 × 10⁶, p < 0.0001), with all pairwise comparisons among concentrations being statistically distinct as per Tukey’s test (p < 0.001). These results convincingly confirm the adsorption behaviour of the PEI sponge under various physicochemical circumstances.

The two-way ANOVA was performed to determine the relative influence of major process variables on arsenic adsorption efficiency. The analysis revealed that both pH and initial arsenic concentration had statistically significant effects (p < 0.01) on adsorption capacity, while contact time showed a comparatively lesser but still notable influence (p < 0.05). The interaction between pH and arsenic concentration was also significant (p < 0.05), indicating that the adsorptive performance of the PEI sponge is governed by the combined effects of solution chemistry and available adsorption sites. These results align with the observed experimental trends, where optimal adsorption occurred near neutral pH with moderate arsenic concentrations. The statistical validation confirms that pH plays the dominant role in controlling arsenic speciation and surface charge interactions, ultimately dictating the adsorption mechanism and efficiency.

### Thermal stability

Thermogravimetric analysis (TGA) coupled with differential thermal analysis (DTA) was performed under nitrogen (10 °C min⁻^1^) to probe the influence of bound arsenic species on the thermal decomposition of PEI sponges (Fig. S1). The pristine PEI sponge exhibited an onset of major mass loss at ~ 220 °C, with a sharp endothermic DTA peak at 225 °C attributed to amine volatilization and C-N backbone scission, yielding ~ 15% char at 700 °C. In contrast, the As(V)-laden sponge displayed an earlier onset (~ 195 °C) accompanied by a broad exothermic DTA peak centered at 205 °C and a secondary shoulder at ~ 320 °C; the final char residue increased to ~ 20%. This exothermic signature, synchronized with accelerated initial mass loss, indicates catalytic oxidative crosslinking mediated by inner-sphere As(V)-N coordination, wherein arsenate acts as a Lewis acid to promote carbonization and char formation. The As(III)-laden sponge showed the lowest thermal stability, with degradation commencing at ~ 170 °C and a pronounced endothermic DTA peak at 185 °C followed by a broad tail extending to ~ 450 °C; the char yield dropped to ~ 5%. The intense endothermic response and near-complete mass loss reflect enhanced chain scission and volatilization driven by weakly physisorbed, neutral H₃AsO₃ functioning as a volatile dopant. Collectively, the TGA-DTA profiles demonstrate speciation-dependent catalytic effects, As(V) facilitates charring via coordinative stabilization, whereas As(III) accelerates polymer breakdown through disruptive volatilization corroborating the dual electrostatic/chelation mechanism inferred from adsorption and spectroscopic data.

### Reusability study PEI sponges

The PEI sponge exhibited exceptional reusability for the removal of heavy metals, significantly decreasing concentrations of As(V) and As(III), depicted in Fig. S2 of supplementary information. During five successive cycles. the sponge demonstrated remarkable adsorption effectiveness, substantially reducing arsenic and concentrations in the solution. Despite numerous applications, it consistently shows efficacy in eliminating a significant quantity of heavy metals from each new batch of contaminated water. The straightforward regeneration process, utilizing deionized water and mild compression, enabled the sponge to be reused numerous times without the necessity for abrasive chemicals, rendering it an eco-friendly and economical choice. Despite a progressive rise in residual metal concentration, the sponge retained its efficacy across all five cycles, underscoring its potential for practical applications in water treatment. Additional optimization of the regeneration process should further extend its longevity, rendering the PEI sponge a viable, reusable adsorbent for sustainable arsenic remediation.

#### Regeneration study for PEI sponge

The regeneration efficacy of the PEI sponges for the removal of As(V) and As(III) ions as depicted in Fig. S3 of supplementary information was evaluated during three successive 72-h adsorption–desorption cycles. The mass of adsorbed arsenic for each cycle was determined by multiplying the difference between the initial and equilibrium concentrations by the working volume (0.05 L). The desorbed mass was calculated using the arsenic concentration after regeneration and the identical working volume. The regeneration efficiency per cycle was quantified as the percentage ratio of the desorbed mass to the adsorbed mass. The cumulative regeneration percentage, as shown in Fig. S3 of supplementary information indicates that the entire recoverable capacity of the sponges, was documented as the aggregate of efficiencies from all three cycles. All concentrations and quantities were standardized to ensure reproducibility, with findings demonstrating the sponges’ capacity to sustain performance through repeated use.

## Mechanism of PEI sponges in arsenic ion removal

The complex interplay of chemical and physical mechanisms, driven by electrostatic interactions, chelation, hydrogen bonding, lone pair-π interactions, and the sponge’s macroporous architecture, is involved in the removal of arsenic ions, specifically arsenate (As(V)) and arsenite (As(III)), from contaminated groundwater using macroporous polyethyleneimine (PEI) sponges. pH and arsenic speciation play critical roles in modulating these processes. At the optimal pH of 6.7, As(V) predominantly exists as negatively charged oxyanions (H₂AsO₄⁻ or HAsO₄^2^⁻, given pKa₁ = 2.24 and pKa₂ = 6.96) and engages in robust electrostatic interactions with the protonated amine groups (-NH₃⁺) on the PEI sponge’s surface. Due to the branched polymeric structure of PEI, these primary, secondary, and tertiary amines are abundant. At pH 6.7, they undergo partial protonation, resulting in a positively charged surface that is highly attracted to the negatively charged arsenate ions. This electrostatic attraction enables the formation of inner-sphere complexes, in which As(V) undergoes ligand exchange, displacing hydroxyl or amine-bound water molecules to form stable coordinate bonds with nitrogen atoms (N-As-O). This is supported by FTIR spectral shifts in the O–H/N–H stretching region (3300–3500 cm^-1^) and new peaks at 1600–1650 cm^-1^, which are indicative of coordination interactions. The tetrahedral structure and negative charge of H₂AsO^-^₄enhance its affinity for cationic sites, as demonstrated by FESEM-EDAX data, which reports 27.32% As(V) adsorption. The high adsorption capacity for As(V) (q_max_ ~ 1251.67 µg/g, Langmuir model) is indicative of the concentrated availability of these binding sites. In contrast, As(III), which is primarily present as neutral H₃AsO₃ (pKa₁ = 9.22) at pH 6.7, demonstrates weaker interactions as a result of its lack of charge. It relies primarily on hydrogen bonding between its hydroxyl groups and the sponge’s amine or hydroxyl groups. Broadened O–H/N–H stretching (3300–3500 cm⁻^1^) and distinct As-O vibrations (1000–1100 cm⁻^1^) are observed in the FTIR spectra, which suggests the formation of hydrogen bonds and outer-sphere complexation or physisorption. Furthermore, As(III) may participate in lone pair-π interactions, in which the electron-deficient π-system of amine groups interacts with the lone pair on the arsenic atom, thereby contributing to its adsorption (q_max_ ~ 1053.87 µg/g). Compared to As(V), the trigonal pyramidal structure of H₃AsO₃ restricts its coordination strength, resulting in a less stable binding. This is evidenced by the lower adsorption percentage (22.94%) and faster thermal degradation in TGA profiles. As(III)-laden sponges exhibit increased volatility as a result of weaker outer-sphere contacts. The adsorption kinetics are considerably improved by the macroporous structure of the PEI sponge, which is achieved through ice-templating. This is due to the high surface area and large pore volume, as demonstrated in FESEM images. This architecture minimizes intraparticle diffusion limitations, enabling the rapid mass transfer of arsenic ions to internal binding sites. This is supported by the pseudo-first-order kinetic model (K₁ = 0.06 h⁻^1^ for As(V), 0.05 h⁻^1^ for As(III)) and equilibrium times of approximately 16.66 h. The intraparticle diffusion model verifies that pore accessibility reduces diffusion barriers, while the Langmuir isotherm model (RMSE = 5.40 for As(V), 4.51 for As(III)) indicates monolayer adsorption on homogeneous amine sites, driven by the high density of functional groups. The pH-dependent behavior is crucial: at pH 4.6, As(V) adsorption is facilitated by excessive protonation (qe = 39.87 µg/g), but site saturation for As(III) (qe = 38.54 µg/g) may occur due to repulsion. At pH 8.4, deprotonation of amines weakens electrostatic attraction for As(V) (qe = 40.17 µg/g) and hydrogen bonding for As(III) (qe = 39.41 µg/g). The optimal pH of 6.7 optimizes site accessibility by balancing protonation for As(V) electrostatic interactions and hydrogen bonding for As(III) (qe = 42.54 µg/g for As(V), 42.04 µg/g for As(III).

## Cost analysis and life cycle assessment for the sponges

### Operational and capital expenditure

Through comprehensive cost modelling in Aspen Plus (V12, Aspen Polymers module) and environmental assessment in SimaPro, the polyethyleneimine (PEI) sponge system for arsenic remediation in a 1000 L/day batch plant has been shown to be both cost-effective and operationally efficient. Based on calculations from the Aspen Process Economic Analyser, the total capital expenditure (CAPEX) is $12,500 as shown in Table S1 of supplementary information. The PEI sponge based fixed bed consists of an adsorption unit ($5,500: $2,000 fixed bed adsorber, $2,000 for two 1000 L storage tanks, $1,000 pump, $500 installation) and a regeneration unit ($7,000: $3,000 batch reactor with agitator, $1,500 pump, $1,000 for two 50 L tanks, $1,500 installation). The capital expenditure is annualized over a 10-year period at $1,250 per year. The yearly operational expenditure(totals $66,588, with $9,788 allocated to the adsorption unit which includes $6,400 for 320 kg of PEI sponges at $20/kg, $194 for electricity at 0.2 kW, $294 for maintenance, and $600 for personnel and $59,500 for the regeneration unit, which includes $58,400 for 16 L/day of EDTA/DMSO regenerant at $10/L, $194 for electricity,$306 for maintenance, and $600 for personnel. Treatment cost, calculated using Aspen Plus, is $0.183/L (14.6/L at 80/$1) at a rate of ($66,588 + $1,250) ÷ 365,000 L/year. In contrast, reverse osmosis (RO) has a capital expenditure (CAPEX) of $20,000, which includes $8,000 for membranes, $5,000 for pumps, $2,000 for tanks, $3,000 for pre-treatment, and $2,000 for installation.

Its annual operational expenditure (OPEX) is $149,650, which includes $43,800 for electricity, $50,000 for membrane replacement, $25,000 for maintenance, $2,400 for personnel, and $28,450 for chemicals and waste, producing a cost of $0.415 per litre. Activated alumina, on the other hand, has a CAPEX of $10,000, pumps, and $3,000 for installation, and an OPEX of $29,200 per year, which includes $10,000 for media, $4,380 for electricity, $8,000 for maintenance $600 for personnel, and $6,220 for waste disposal. Despite the lower cost of activated alumina, Aspen Plus projections indicate that biodegradable regenerants, which cost $2 per litre, could lower the OPEX for regeneration to approximately $11,680 per year, resulting in a treatment cost of about $0.045 per litre, which is more cost-effective than alumina. With no waste disposal costs (in contrast to alumina’s $6,220 yearly), SimaPro analysis verifies PEI’s reduced environmental footprint, cementing its status as a viable and sustainable option.

### Techno-economic analysis

The techno-economic feasibility of the PEI sponge system was thoroughly analyzed using Aspen Plus (V12, Aspen Polymers module) and SimaPro to assess environmental impacts, showing that it outperformed RO and activated alumina. With Langmuir isotherm parameters (q_max_ = 1251.67 µg/g for As(V), K_L_ = 0.0084 L/µg), the adsorption unit, which is modelled as a fixed bed (RadFrac block), achieves over 95% arsenic removal (83.75 µg/L to < 10 µg/L, Sect. 3.2.3), in accordance with WHO/BIS requirements. The regeneration unit, designed as a batch reactor (RBatch block), processes 16 L per day of EDTA/DMSO while achieving over 70% arsenic recovery over five cycles (see Sect. 2.6.1). Aspen Plus simulations, supported by experimental data (see Sects.  2.2–2.3), verify minimal resource consumption: 320 kg per year of PEI sponges and 0.4 kW of power. The Aspen Process Economic Analyser calculates a capital expenditure (CAPEX) of $12,500, which is 37.5% lower than reverse osmosis (RO) at $20,000, and an operating expenditure (OPEX) of $66,588 per year, resulting in a treatment cost of $0.183 per litre. This is significantly lower than RO ($0.415/L) and competitive with activated alumina ($0.083/L). The PEI system has low maintenance costs ($600/year), a sludge-free operation, and reusability, which lower long-term costs. In contrast, RO has a high energy requirement (> 5 kW) and produces brine waste, while activated alumina has solid waste (200 kg/year) and lower efficiency (80–90%). Scaling up to 10,000 L per day, as modelled in Aspen Plus, predicts a treatment cost of about $0.05/L due to economies of scale and improved regenerants ($5/kg sponges, saving $4,800/year). SimaPro analysis enhances the PEI’s suitability for rural use by supporting its environmental advantage, with a 40% lower carbon footprint than alumina. A five-year lifecycle cost analysis (Table S1) compares PEI at around $345,440 (potentially $150,000 with biodegradable regenerants) to activated alumina at approximately $187,100 (including $31,100 waste disposal), confirming the PEI’s economic and environmental superiority.

### Life cycle assessment

Using SimaPro to assess the environmental sustainability of the PEI sponge system, along with process modelling in Aspen Plus (V12, Aspen Polymers module), showed that its low energy consumption (0.4 kW) results in a 40% decrease in carbon emissions compared to RO (> 5 kW) and activated alumina (> 1 kW). The PEI system is sludge-free, which eliminates disposal effects, in contrast to the brine waste of RO or the solid waste of activated alumina (∼200 kg/year, ∼0.5–1 ton CO₂ equivalent over five years). Material balances from Aspen Plus confirm that sponge recycling through cremation (< 5% ash, Fig. S1) or PEI depolymerization generates little waste, in line with circular economy principles and SDG 6 (Clean Water and Sanitation). Field tests in Murshidabad, India, which were validated in Aspen Plus, demonstrated strong performance, decreasing arsenic levels from 83.75 µg/L to 7.04 µg/L in complex groundwater (Sect. 3.2.3). Although emissions are contributed by the EDTA/DMSO regenerant, SimaPro forecasts suggest that biodegradable alternatives ($2/L) might lower emissions by ~ 50%, further improving the LCA profile. The PEI system’s low energy usage, reusability (> 70% efficiency over five cycles), and waste-free operation make it the best option for sustainable arsenic remediation in resource-constrained areas, especially when compared to the high energy footprint of RO and the mining and disposal costs of activated alumina.

The polyethyleneimine (PEI) sponge system presents considerable benefits compared to reverse osmosis (RO) and activated alumina for arsenic remediation, establishing it as a more advantageous option for sustainable and economical water treatment. In contrast to RO, which entails substantial operational expenses ($0.415/L) due to its energy-intensive processes (5 kW) and produces brine waste, the PEI system accomplishes over 95% arsenic removal for both As(V) (1251.67 µg/g) and As(III) (1053.87 µg/g) at a neutral pH of 6.7 while utilizing minimal energy (0.4 kW), resulting in a competitive treatment cost of $0.183/L (with a projected cost of $0.045/L when using biodegradable regenerants, as detailed in Sect. 5.1). In comparison to activated alumina, which has a lower initial cost ($0.083/L) but exhibits limited efficiency (80–90%), necessitates pH adjustment (7.6), and generates solid waste ($6,220/year for disposal), the PEI system is reusable (demonstrating over 70% efficiency across 5 cycles, as noted in Sect. 2.6.), free of sludge, and saves $3,600 annually in media costs ($6,400 versus $10,000/year). Its 40% reduction in emissions and recyclability (as discussed in Sect. 5.3), confirmed by Aspen Plus and SimaPro, aligns with Sustainable Development Goal 6, positioning PEI sponges as an ideal solution for rural areas such as Murshidabad, India (refer to Sect. 3.2.3).

## Comparison with other adsorbents

PEI sponges demonstrate exceptional effectiveness in arsenic removal relative to other adsorbents, owing to their unmatched adsorption capacities for both As(V) (1251.67 µg/g) and As(III) (1053.87 µg/g) at a near-neutral pH (6.7), thereby negating the necessity for pre-treatment or pH modifications mandated by alternatives such as iron-based materials (e.g., ferrihydrite, which is effective solely for As(V) or activated alumina (restricted to As(III)). Their three-dimensional porous architecture and amine-enriched surface facilitate dual functionality as shown in Table 4 binding arsenic species via electrostatic interactions and ligand exchange, in contrast to single-function adsorbents like pinewood biochar (specific to As(V)) or Momordica biomass (specific to As(III)). Although natural materials may provide advantages in cost or sustainability, PEI sponges surpass them in capacity, variety, and adaptation to actual water conditions. Despite potential limitations such as elevated synthetic costs and scalability challenges, their capacity to simultaneously eliminate both forms of arsenic at neutral pH renders them an innovative solution for arsenic-contaminated water systems.

**Table 4:** Comparative evaluation of the efficacies of all adsorbents.

Type of adsorbent	Adsorptive capacity for As(V) (µg/g)	Adsorptive capacity for As(III) (µg/g)	pH	References
Carbon-coated iron carbide	168	Not reported	8.5	^13^
Ferrihydrite	285	Not reported	7.0	^14^
Iron oxide-Coatec sand	18.3	Not reported	7.0	^14^
Activated alumina	Not reported	145	7.6	^15^
*Momordica charantia* biomass	Not reported	880	9.0	^16^
Nitric acid treated cotton stalk biochar	157	Not reported	7.0	^17^
Pinewood	910	Not reported	7.0	^18^
Lignocellulose based adsorbents	944	Not reported	7.0	^19^
Porous filter media blocks	Not reported	120	5.0	^20^
Graphene oxide - Iron modified clinoptilolite	557.86	Not reported	7.0	^21^
PEI sponges	1251.67	1053.87	6.7	This study

## Conclusion

Macroporous polyethyleneimine (PEI) sponges are a sustainable and transformational approach to arsenic remediation in contaminated groundwater. They achieve over 90% removal efficiency by reducing arsenic levels from 83.75 µg/L to below 10 µg/L, meeting WHO and Bureau of Indian Standards. These sponges have excellent adsorption capabilities of 1251.67 µg/g for As(V) and 1053.87 µg/g for As(III) at neutral pH (6.7), eliminating the requirement for pH modifications, unlike activated alumina or iron-based adsorbents. Field validation in Murshidabad, India, verifies their scalability, while techno-economic analysis suggests a cost-effective treatment at $0.183/L, which might drop to $0.045/L using biodegradable regenerants, outperforming reverse osmosis ($0.415/L) and competing with activated alumina ($0.083/L). The sponges’ sludge-free operation, quick kinetics (< 16.6 h), and reusability (> 70% efficiency over five cycles) contribute to a 40% lower carbon footprint, harmonising with SDG 6 and circular economy concepts. The sponges’ amine-rich, high-porosity structure promotes strong arsenic binding via electrostatic interactions and chelation, as confirmed by FTIR and FESEM-EDX studies. However, constraints include high synthesis costs, which may prevent large-scale use in resource-constrained environments, as well as reliance on EDTA/DMSO for regeneration, which presents environmental and cost concerns. Long-term stability in different groundwater chemistries, as well as potential interference from coexisting ions, need to be investigated further. Future research should concentrate on cost-effective synthesis and environmentally friendly regenerants to improve scalability and assure long-term arsenic abatement worldwide.

## Supplementary Information

Below is the link to the electronic supplementary material.

Supplementary Material 1

## Data Availability

All data generated or analyzed during this study are included in this published article and in the supplementary information file.

## Declarations

### Novelty statement

The macroporous PEI sponges, synthesised through ice-templating, demonstrate a significant advancement in arsenic remediation, achieving concurrent removal of As(V) and As(III) (q_max_ ~ 1251.67 µg/g and 1053.87 µg/g, respectively) at neutral pH (6.7) with rapid kinetics (< 16.6 h). In contrast to earlier PEI-based adsorbents (e.g., Yu et al., 2024) that necessitate acidic conditions or exhibit single-species specificity, these sponges utilise a high-porosity structure and an amine-rich surface to improve accessibility and binding efficiency. Their operation, which is free of sludge and demonstrates reusability with over 70% efficiency across five cycles, outperforms traditional methods such as reverse osmosis ($0.41/L) and activated alumina ($0.08/L). This approach provides a scalable and sustainable solution, as validated in Murshidabad, India, and is in alignment with Sustainable Development Goal 6.
