# Supplementary material for: Reusable macroporous polyethyleneimine sponges for sustainable removal of dual-arsenic species: mechanistic and life cycle assessment
Source: Sci Rep. 2026 Apr 10;16:16749. doi: 10.1038/s41598-026-45664-1 (PMC13223241; doi:10.1038/s41598-026-45664-1)
Supplement: Supplementary file 1 — Supplementary Material 1 [file 41598_2026_45664_MOESM1_ESM.docx]

**Supplementary Information**

**Reusable Macroporous Polyethyleneimine Sponges for Sustainable Removal of Dual-Arsenic Species: Mechanistic and Life Cycle Assessment**

**Fig.S1** Thermogravimetric analysis for PEI sponges


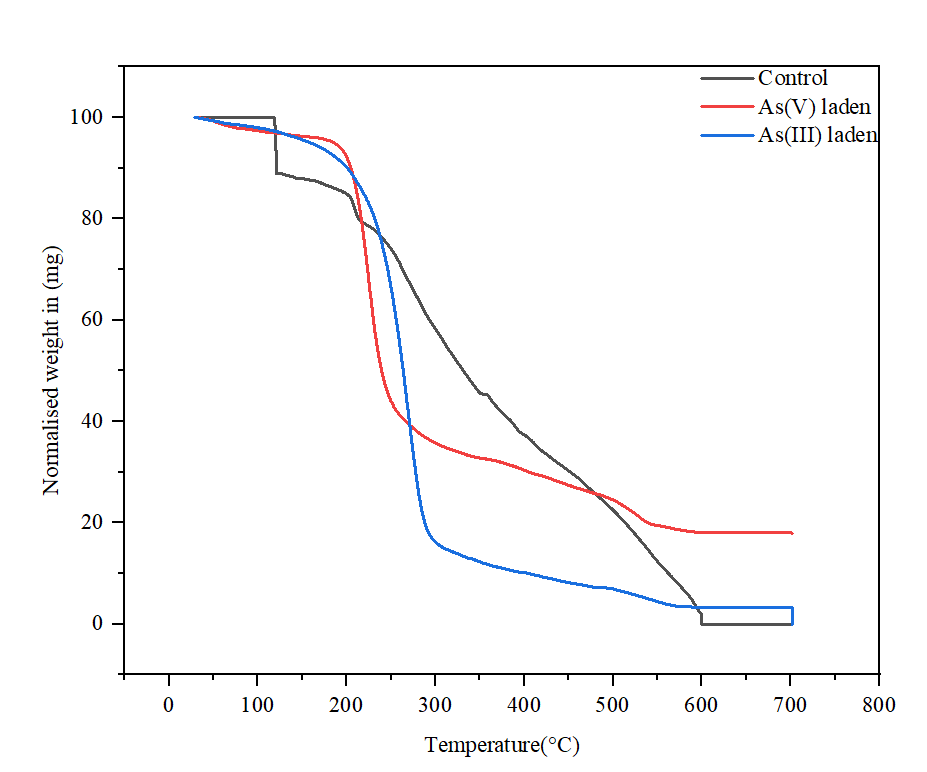


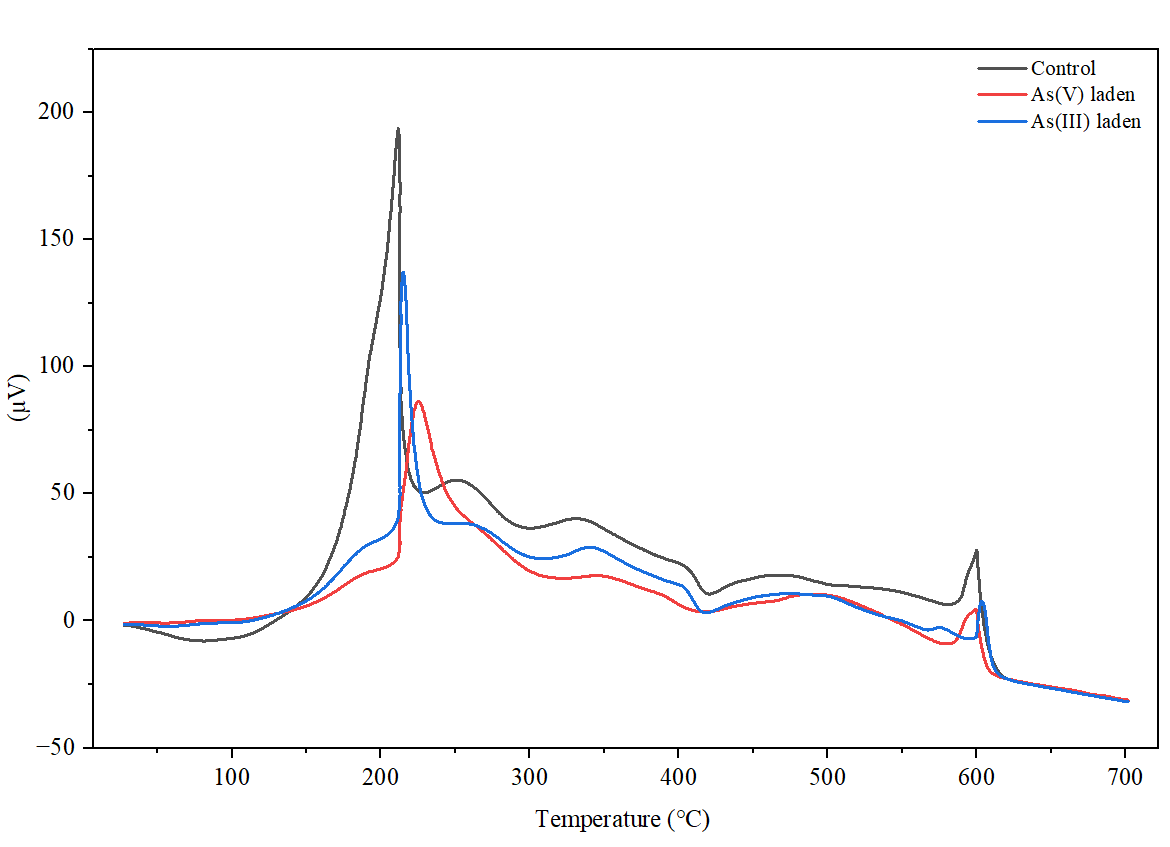


**Fig S2**. Reusability of the PEI sponges


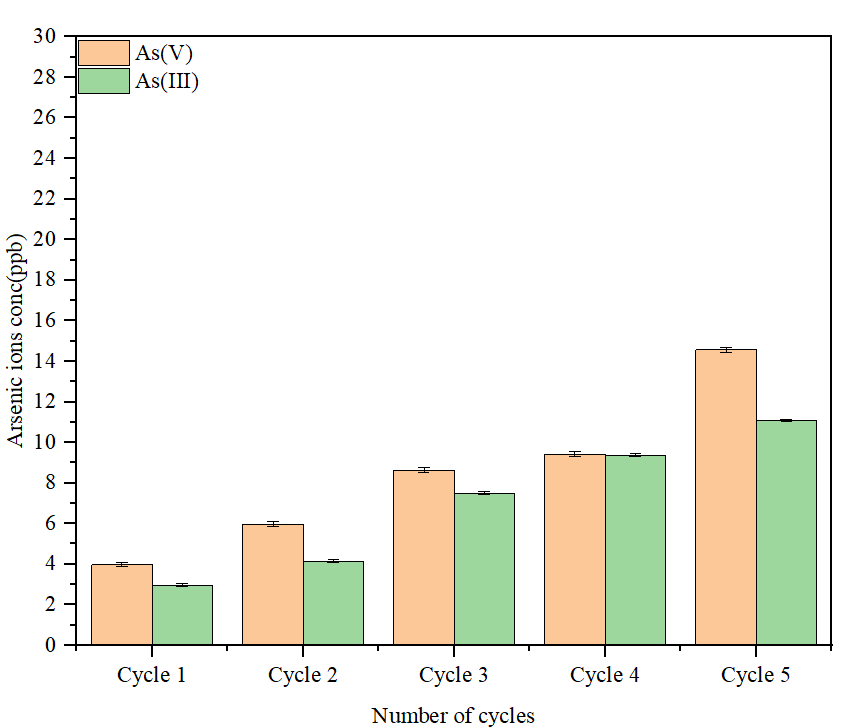


**Fig S3.** Regenerability study of PEI sponges


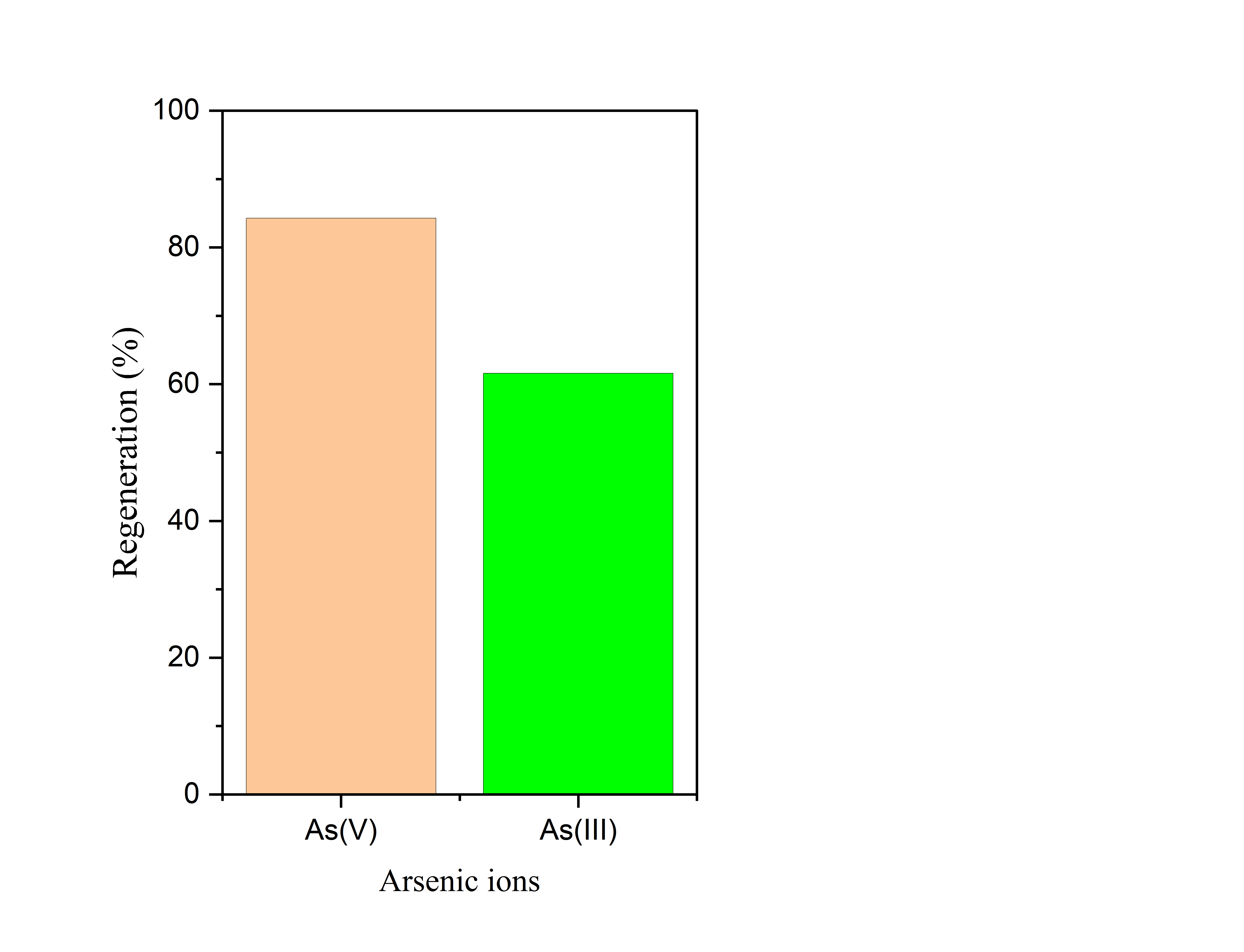


| Table S1. Cost Analysis for PEI Sponge System along with Conventional Methods |
| --- |

| **Parameter** | **PEI Sponge System** | **Reverse Osmosis (RO)** | **Activated Alumina** |
| --- | --- | --- | --- |
| **CAPEX Breakdown** |  |  |  |
| Adsorber/Reactor | $2,000 (Fixed bed) | $8,000 (Membranes) | $4,000 (Fixed bed) |
|  | $3,000 (Batch reactor) |  |  |
| Storage Tanks | $2,000 (2 x 1000 L) | $2,000 (2 x 1000 L) | $2,000 (2 x 1000 L) |
|  | $1,000 (2 x 50 L) |  |  |
| **Total CAPEX** | $12,500 | $20,000 | $10,000 |
| **Total OPEX** | $66,588/year | $149,650/year | $29,200/year |
| **Treatment Cost ($/L)** | $0.183 (proj. $0.045) | $0.415 | $0.083 |

**Table S2.**  Pseudo-second order kinetics for arsenic under acidic, neutral and basic pH

| **Heavy metal ion** | **pH** | ***q*_e_ (µg/g)** | ***K*_1_ (hr^-1^)** | **RMSE** |
| --- | --- | --- | --- | --- |
| As(V) | 4.6 | 48.39 | 0.03 | 3.28 |
|  | 6.7 | 57.21 | 0.06 | 1.23 |
|  | 8.4 | 53.16 | 0.04 | 3.16 |
| As(III) | 4.6 | 46.03 | 0.02 | 3.10 |
|  | 6.7 | 55.37 | 0.05 | 1.17 |
|  | 8.4 | 51.22 | 0.03 | 3.02 |

**Table S3** Pseudo-second order kinetics for arsenic removal at higher concentrations

| Arsenic species | Initial Concentration (µg/L ) | Parameters | | RMSE |
| --- | --- | --- | --- | --- |
|  |  | ***q*_e_ (µg/g)** | ***K*_1_**  **(h^-1^)** |  |
| As(V) | 30 | 66.94 | 0.05 | 4.98 |
|  | 50 | 94.41 | 0.05 | 5.01 |
|  | 70 | 116.37 | 0.06 | 7.60 |
|  | 100 | 136.21 | 0.04 | 8.14 |
|  | 120 | 187.04 | 0.05 | 9.09 |
| As(III) | 30 | 61.38 | 0.05 | 4.61 |
|  | 50 | 90.36 | 0.06 | 5.45 |
|  | 70 | 112.53 | 0.05 | 7.04 |
|  | 100 | 131.24 | 0.05 | 8.71 |
|  | 120 | 182.48 | 0.06 | 9.84 |

**Table S4** Surface charge analysis of PEI sponge

| pH | Zeta Potential  (mV) | Surface Charge | Dominant Arsenic Species |
| --- | --- | --- | --- |
| 4.6 | 38.2 ± 1.1 | Strongly positive | H₃AsO₄ (As(V)), H₃AsO₃ (As(III)) |
| 6.7 | 22.5 ± 0.9 | Moderately positive | H₃AsO₄ (As(V)), H₃AsO₃ (As(III)) |
| 8.4 | 5.8 ± 1.3 | Near-neutral | H₃AsO₄ (As(V)), H₃AsO₃ (As(III)) |

**Fig**. **S4** (a) Non-linear Langmuir isotherm for As(V) ,(b) As(III) removal.


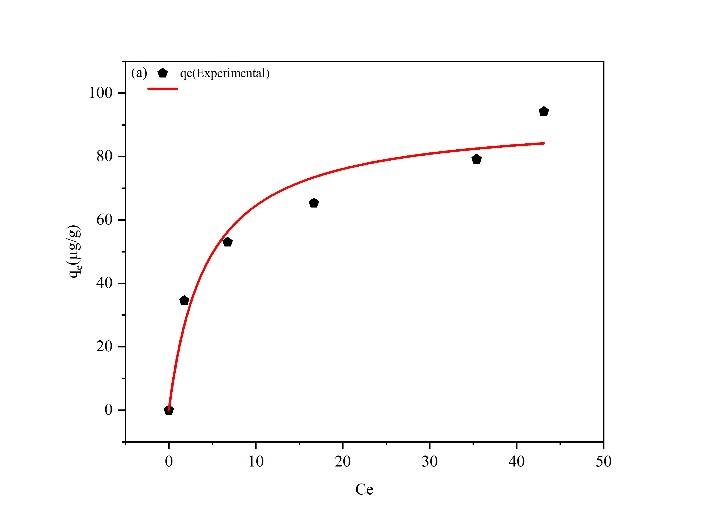

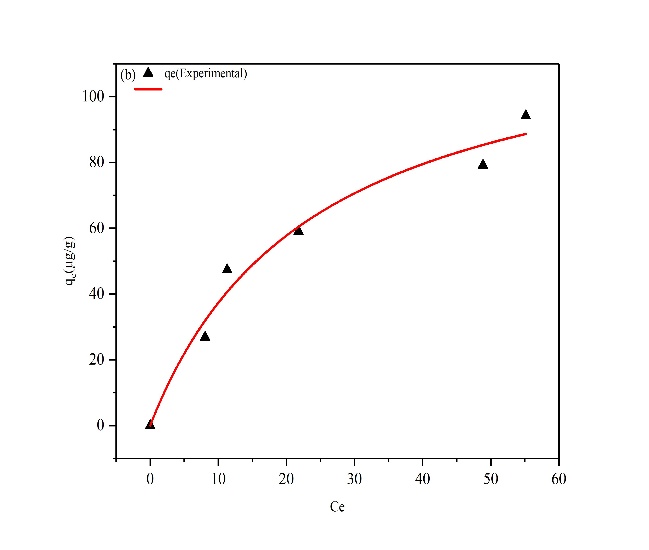


**Fig. S5** N₂ Adsorption-Desorption Isotherm


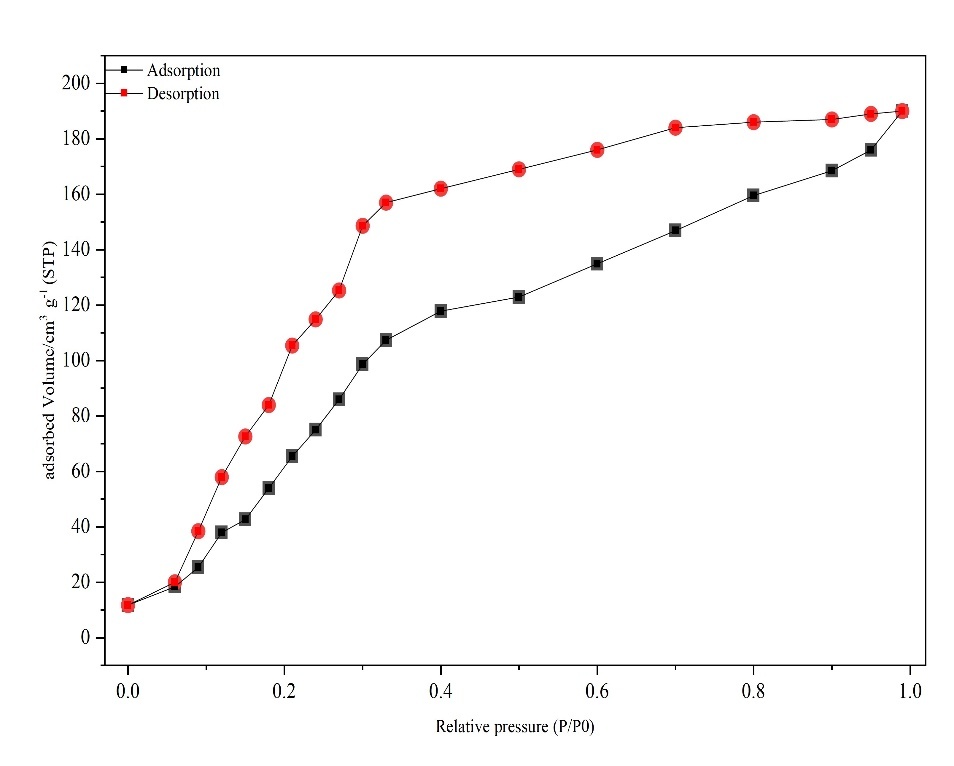


**Table S5** BET analysis for PEI sponges

| Sample | BET Surface Area (m² g⁻¹) | Pore Volume (cm³ g⁻¹) | Average Pore Diameter (nm) | Observation |
| --- | --- | --- | --- | --- |
| Control (Unloaded PEI sponge) | 52.0 | 0.42 | 62 | Hierarchical meso-macroporous structure; facilitates rapid diffusion. |
| As(V)-laden PEI sponge | 41.5 | 0.34 | 58 | Slight decrease due to pore occupation and partial blockage by As(V) complexes. |
| As(III)-laden PEI sponge | 39.2 | 0.31 | 56 | Further reduction attributed to neutral As(III) penetration and multilayer accumulation within pore walls. |
